# Perceptions of Children and Young People in England on the Smokefree Generation Policy: A Focus Group Study

**DOI:** 10.1093/ntr/ntae300

**Published:** 2024-12-17

**Authors:** Nathan P Davies, Rachael L Murray, Tessa Langley, Joanne R Morling, Manpreet Bains

**Affiliations:** Nottingham Centre for Public Health and Epidemiology, University of Nottingham, Nottingham, UK; Nottingham Centre for Public Health and Epidemiology, University of Nottingham, Nottingham, UK; SPECTRUM Consortium, Edinburgh, UK; Nottingham Centre for Public Health and Epidemiology, University of Nottingham, Nottingham, UK; SPECTRUM Consortium, Edinburgh, UK; Nottingham Centre for Public Health and Epidemiology, University of Nottingham, Nottingham, UK; NIHR Nottingham Biomedical Research Centre, Nottingham University Hospitals NHS Trust with the University of Nottingham, Nottingham, UK; Nottingham Centre for Public Health and Epidemiology, University of Nottingham, Nottingham, UK

## Abstract

**Background:**

Modeling shows smokefree generation (SFG) policies could effectively reduce smoking rates by banning tobacco sales to those born after a specific year. Little is known about how young people perceive the legitimacy and impact of the planned SFG policy in England.

**Methods:**

We conducted seven semi-structured focus groups with 36 participants aged 12–21 (mean = 15) in England over video call and in person. Twenty-one participants were female and 15 male. Participants were purposively sampled to include those from areas of greater deprivation and for use of tobacco or e-cigarettes. Data was analyzed using the framework approach.

**Results:**

Participants expressed broadly negative perceptions toward tobacco and its manufacturers. Most participants supported SFG policy goals and its focus on freedom from addiction and harm; some believed it should also encompass electronic cigarettes. Many believed the law would only be successful if it included stringent enforcement, accompanying tobacco licensing, and input from young people. A minority raised concerns about the loss of freedom to purchase tobacco.

**Conclusions:**

Communication of the freedom-giving nature of SFG is likely to resonate with many young people. Enforcement, communication, and involvement of young people in SFG should be considered carefully to maximize policy impact.

**Implications:**

The smokefree generation (SFG) policy’s potential to offer freedom from addiction and disease can resonate with young people. Its effectiveness could be maximized through targeted enforcement in areas with high youth smoking rates and low adherence to age-of-sale laws, and through the introduction of additional policies that offer restrictive licensing of tobacco retailers. A phased approach to SFG, initially covering tobacco and later incorporating e-cigarettes as smoking prevalence declines, could balance reducing youth vaping and harm reduction; future research could investigate optimal policy conditions for this approach.

## Introduction

Preventing smoking initiation and addiction in children and young adults has long been crucial to the reduction of smoking rates.^1^ Evidence from the first coronavirus disease 2019 lockdown in England found a 25% increase in smoking prevalence among 18–34-year-olds.^2^ This indicates that long-term decline in smoking in young adults in countries with strong tobacco control should not be taken for granted.

One option for reducing smoking among young people is to raise the minimum legal age of sale for tobacco products (MLSA) beyond 18 or the local age of adulthood.^3^ In 2019, the United States implemented a federal law setting an MLSA of 21 (Tobacco 21).^4^ Other nations have reportedly introduced an MLSA of at least 20.^5^ A systematic review found introducing Tobacco 21 in the United States likely reduced smoking rates among older adolescents and young adults.^6^ However, the policy’s impact was inconsistent in early adopter areas, such as New York and California, which had issues with communication, design, and enforcement of the law.^7–9^ Qualitative research into Tobacco 21 suggests that while some young people are supportive, some disagree with its perceived subversion of the traditional age of adulthood, and some doubt its effectiveness.^10,11^

There are other age-of-sale policy options. A smoke-free generation policy (SFG), also known as a tobacco-free generation policy, bans the sale of tobacco products to anyone born after a specific year. These policies overcome a key limitation of age-based age-of-sale policies, which still convey the message that tobacco use is socially acceptable and a rite of passage into adulthood.^12,13^ Simulation modeling for New Zealand Aotearoa,^14,15^ Singapore,^16,17^ and the United Kingdom^18^ all find that, over long timeframes, SFG is likely to be one of the most effective policies for reducing harm from tobacco.

Several areas have proposed SFG policies, including Tasmania (Australia), Finland, Malaysia, and Denmark.^19–22^ However, these proposals have foundered under changes of government policy, or the possibility or reality of legal challenges. New Zealand Aotearoa’s 2022 SFG policy was to form part of a tobacco endgame strategy alongside denicotinization and tobacco outlet reduction.^23^ In 2024, the relevant laws were reversed by a newly elected coalition Government, which argued repeal was necessary to fund separate tax cuts.^24^

Brookline, a small town in Massachusetts, United States, successfully defended a local bylaw introducing an SFG ban on nicotine product sales at its state Supreme Court.^25^ Several other small US towns have subsequently passed nicotine-free generation laws.^26^

In March 2024, the Conservative UK Government introduced the Tobacco and Vapes Bill, which included ban on all tobacco product sales to those born in 2009 onwards.^27^ Electronic cigarettes (e-cigarettes) were not included under SFG, but measures to restrict their distribution to young people, such as new powers to regulate flavors, packaging, and product requirements, were included in the Bill.^27^

Despite support, the Bill failed to become law before the May 2024 general election. The Labour Government elected in July 2024 has declared its intention to re-introduce SFG in its term of office.^28^

To date, there has been no qualitative study with young people in England who will be affected by SFG. This type of research can provide important context to guide enforcement and to understand how SFG may affect social norms around tobacco, both of which are key considerations when implementing effective laws on tobacco age-of-sale.^29^ It can help identify potential barriers to policy implementation, such as misconceptions about the policy, which messages are most likely to resonate, or unintended consequences, which may not be apparent to policymakers.^30^ Furthermore, it is a central tenet of public health ethics to directly engage with people who will be affected by public health policy.^31^ This study aims to address this evidence gap by exploring the perceptions of children and young people in England toward the planned SFG law.

## Methods

### Overview

We conducted focus groups with a range of young people who either will be directly affected by SFG in England or who were at an age where they could conceptualize SFG affecting themselves and their peers. We sought to understand their views on the tobacco product SFG proposals in England, and, because of the ongoing debate on whether to include e-cigarettes in new tobacco control regulations, perceptions of how SFG could or should relate to e-cigarette use.

### Design and Epistemological Approach

While interviews can enable deep exploration of individual perspectives,^32^ individual interviews with adults can be intimidating. Focus groups can help overcome the adult–child power dynamic and create a more relaxed environment for giving franker responses.^33^ Furthermore, our young public advisors indicated that focus groups were preferred. Given the inherently social nature of youth smoking, the research was underpinned by a constructionist approach. Participants’ words were given meaning and interpreted through the context of their social and wider environments.^34^ The lead researcher, ND, a male public health specialist with experience in qualitative research, was trained in focus group methodology specifically for children and young people.^35^ Those analyzing the data undertook bracketing, where researchers intentionally recognized, wrote down, and set aside pre-conceptions to approach the data with an open perspective.^36^ The COREQ framework has been used to support transparent reporting.^37^

### Sampling and Recruitment

Our inclusion criteria incorporated children and young people living in England aged between 11 and 21, who were those who would be directly affected by SFG, and older children and young people who would not be affected but who could conceive of the law affecting their age group. An SFG law will affect those who have already tried smoking, those who may try smoking in the future, those who use e-cigarettes but may or may not also use tobacco in future, and those who will never try any nicotine products, but whose friends and family are affected by tobacco. Therefore, we began by sampling for maximum variation across age, gender, ethnicity, and region, before purposively sampling for theoretical saturation of groups that were underrepresented in our data, such as certain age groups or for tobacco or e-cigarette use. We purposively sampled most participants to live in areas of greater deprivation to support consideration of those most affected by health inequalities.

We recruited participants through the support of a range of organizations working with children and young people, such as schools, colleges, youth groups, and education and employment charities. These organizations purposively approached young people based on our inclusion criteria. Participant information sheets were provided in advance by gatekeeper organizations and the lead interviewer also verbally discussed them with participants ahead of focus groups. Participants completed an online or paper consent form before focus groups commenced and parental permission was sought for those aged under 16.

### Data Collection

Participants completed a paper or online questionnaire reporting demographic information, including any smoking and e-cigarette history. Based on prior literature,^38^ the UK government’s SFG command paper^39^ and the COM-B framework^40^ we developed a topic guide (Supplementary File 1) covering (1) personal experiences of tobacco and e-cigarette use (2) the concept of SFG (3) the implementation of SFG. Participants were offered a 10 GBP shopping voucher to compensate for their time.

ND conducted focus groups in-person and over MS Teams, depending on organizational and participant preference. Participants were in groups with similarly aged children and young people. There was a maximum age gap of 3 years between participants. For safeguarding reasons, an adult from gatekeeping organizations was present for focus groups with participants under the age of 18. They sat away from the main group or had their camera switched off and were asked not to speak. Informal conversations took place before recording to explain the process, to give an opportunity for questions, to set boundaries of confidentiality, and for participants to say a bit about themselves and feel comfortable in the group.^41^ All those under 18 were in a group with others they knew. Young people were reassured that their views would be reported completely confidentially and there was no “right answer” to questions. All participants were given the opportunity to contribute to each section of the discussion topic to enable all views to be heard. Focus groups lasted between 26 and 36 minutes (mean = 32 minutes) and were recorded via MS Teams or recording device. Initial transcription was made through the University of Nottingham’s automated transcription service and corrections were made by ND. No significant new themes or ideas were noted in the sixth or seventh focus groups and so no further recruitment took place.

### Data Analysis

Transcripts were read several times to support data familiarization. Initial codes, themes, and subthemes were generated inductively by ND with the use of NVivo 12.^42^ Three of seven transcripts were double-coded by MB to provide triangulation and enhance the credibility of the analysis.^43^ An initial thematic framework was developed to index and chart data with the incorporation of a constant comparative method to ensure all data was considered and the framework approach was used to structure final theming.^44^ Themes were reviewed by ND and MB at various points in the analysis before being finalized.

### Ethical Approval

Ethical approval was granted by the Faculty of Medicine and Health Sciences Research Committee at the University of Nottingham (reference FHMS 39-1023).

### Public Involvement

Three separate groups of public advisors aged 12–21 (*n* = 18) provided advice on inclusion criteria, recruitment methods, and the topic guide (see Supplementary File 2 for further details).

## Results

Seven focus groups were conducted comprising 36 participants aged 12–21 from across England. Demographic details are reported in Table 1. Some participants made an informed decision not to share their postcode (*n* = 7), smoking status (*n* = 2), or other ethnicity (*n* = 3). Fifty-eight percent of the sample were female, 47% were aged 12–14 and 61% were White British. Fifty-three percent of the sample had tried tobacco and 82% had tried e-cigarettes. Fifty-nine percent of participants were from the most deprived quintile of English postcodes.

**Table 1.** Participant demographics

Characteristics	*n*
**Gender**	
Female	21
Male	15
Other	0
**Age (years)**	
12–14	17
15–17	14
18–21	5
**E-cigarette use**	
Never	6
Tried once or twice	3
Current use	22
Missing	2
**Tobacco use**	
Never	16
Former use	9
Tried once or twice	9
Current use	7
Missing	2
**Postcode deprivation quintile (1 = most deprived)**	
1	10
2	7
3	7
4	2
5	3
Missing	7
**Ethnicity**	
White British	22
Gypsy Roma	4
Mixed background	4
Black	2
White Other	1
Any other background	1
Missing	3

### Thematic Framework

Three inductive themes relating to the SFG policy were identified. The themes are: (1) Escape from harmful addiction (2) Impact of SFG is not guaranteed. (3) Give us protection and a voice. We identified some differences according to the use of tobacco or e-cigarette products and some differences between age groups, but we did not identify differences between genders. Where supporting quotes are provided, brackets signify (gender, age, smoking/e-cigarette status). For smoking/e-cigarette status, T = tried, F = former, R = regular, N = never, S = smoked tobacco, and V = use of vapes. The thematic framework is provided in Supplementary File 3.

### Escape from Harmful Addiction

Most participants supported the aims of SFG in its broadest sense. They conveyed a sense of enthusiasm towards the idea of being part of a SFG. This appeared to be related to negative views of tobacco and the tobacco industry, especially toward the industry’s methods of attracting young purchasers. Both those who had tried cigarettes and those who had not expressed physical revulsion toward smoked tobacco, such as *“Eughh, fags are vile.”* (M17, TS/RV, FG2), with just one participant expressing positive sentiment.

Several participants also expressed feeling helpless about the harm that smoking exacted on their families, like the participant who said, “*We were like really worried about (mother’s smoking). And she said that she was gonna try and stop, but she really hasn’t. And it must be difficult because it’s not something that she really talks to us much about*.” (F13, NS/NV, FG1) Others related their support to the difficulties they had quitting e-cigarettes or smoking and described how the feeling of being addicted took a toll on their lives. Several of these participants expressed the idea that SFG supported positive freedom *from* addiction to tobacco. There was a recognition that making a “decision” to smoke whilst a young adult was far less free than it appeared, with one participant saying, *“It’s a good idea. Because young people can get addicted easily. At the age of 18, it’s too hard for them to make a decision.”* (M18, NS/TV, FG2).

A small minority of participants, mostly those who currently smoked tobacco or used e-cigarettes, suggested SFG would jeopardize personal liberties, bodily autonomy, or contravene the convention of 18 being the age of adulthood. As one participant put it, *“Let’s say if you wanna start cigarettes then you can smoke cigarettes. Like, it’s really your life if you want to ruin it.”* (M14, RV/TS, FG3). Another small minority of participants, who were current users of e-cigarettes, indicated apathy toward SFG, with several using phrases similar phrases to “I *don’t really care, to be honest. They can do what they want.”* (F14, RV/NS, FG3). Further probing questions revealed many of this group had little interest in smoking tobacco and were more interested in how e-cigarettes would be regulated.

### Impact of SFG is not Guaranteed

Participants had nuanced discussions on how effective SFG would be in reducing smoking rates, rarely dismissing it completely or predicting total success. The minority of participants against SFG on philosophical grounds were typically very skeptical of the effectiveness of SFG. Some expressed reservations the policy could backfire, suggesting that making tobacco sales illegal would increase its appeal amongst young people and lead to increased smoking rates and law-breaking. *“It’s just gonna cause more illegal activity because there’s more people giving them illegally... I don’t think there’s any point of doing anything with the cigarettes or the vapes because it’s just going to cause more havoc for the government.”* (F14, TS/RV, FG1).

Several drew upon personal experiences of underage purchasing to envisage that retailers and young people (including themselves) would find a way around the regulations through parents or by identifying retailers happy to break the rules. However, many focus group conversations converged on a broad agreement that SFG would not reduce smoking amongst those who were already addicted, but would prevent young nonsmokers from taking up smoking—the central argument for SFG implementation. The sentiment *“For new generations, yeah (the policy will work) but this lot now, no”* (M14, NS/RV, FG4) was commonly expressed.

Other participants often expressed confusion that e-cigarettes were not being included in the SFG proposal, given what they perceived to be the greater problem of e-cigarette use among young people. Two participants felt SFG may lead to greater e-cigarette uptake. This appeared to be linked to significant concerns around the health risks of e-cigarettes, largely centered around lack of knowledge of future damage; many shared the view *“you don’t know the long-term effects (of e-cigarettes)”* (F20, TS/RV, FG7). Participants were largely unconvinced that e-cigarettes were safer than tobacco, despite some awareness e-cigarettes are promoted as a quit aid for adults in England.

Younger participants had incomplete knowledge of existing age-of-sale laws, with many suggesting that the age of sale for cigarettes was 21 or that the age of sale for e-cigarettes was 16, suggesting communication of a new SFG policy may be starting from a low knowledge base.

### Give us Protection and a Voice

Many participants argued that retailers who breached the rules should face the harshest possible penalties, including jail sentences. There was considerable worry that some retailers would seek to bypass the law by existing methods such as covert under-the-counter sales. This was related to a perception from those who had tried or used tobacco or e-cigarettes while under the age of sale perceived tobacco products were currently relatively straightforward to access; one participant said*, “In [area] you can buy anything... Just go to shop, say I want that, they give it to you. They don’t ask for nothing. I recommend you just shut down every shop in [area]”* (M15, FS/RV, FG6). Methods ranged from supply or theft from older friends and family members, picking products up from the street, and direct purchases from retailers.

Participants reported young people needed better protection from retailers who freely sold to underage persons, although one young person expressed positive sentiment toward these retailers. For these reasons, the idea of licensing tobacco retailers and reducing the number of outlets that could sell cigarettes resonated with young people. There was a particular focus on educational settings; one participant said, “*Smoking shops and vaping shops should not be allowed near schools”* (F13, NS/NV, FG1).

There was a plurality of perspectives on how SFG should communicated by governments. This ranged from a strong focus on the smokefree vision to a strong focus on the health impact of tobacco, to the idea that significant government communication could backfire because young people would rebel against the perception of being told what to do. Participants agreed children and young people should be involved in decision-making and implementation of SFG, especially in the design of the communications and providing insight into how tobacco is currently obtained underage. This was justified as being both the right thing to do and to provide unique perspectives into the lives of their peers; one participant said, *“I think (youth involvement) is a really good and important idea because I think it’s important that young people can sort of feel they are the smokefree generation”* (M17, NS/NV, FG7).

## Discussion

### Summary of Findings

Our findings offer insights into the perspectives of children and young people in England on SFG, including a strong representation of participants living in areas of higher deprivation and those with experience of tobacco use or e-cigarette use.

However, the support for SFG is nuanced. While participants appreciate the vision of a smokefree generation, many doubt it will be effective without robust enforcement mechanisms. This skepticism highlights young people understand policy implementation challenges, particularly in areas of higher deprivation where underage sales are prevalent. Their advocacy for licensing fewer tobacco retailers indicates a belief that reducing availability is essential for the policy’s success, aligning with evidence that limited access can decrease youth smoking rates.

### Implications

We found that most participants of all demographics and experiences with tobacco and e-cigarettes expressed a positive conception of the vision and aims of the SFG. They valued positive freedom from addiction, in common with 17- and 18-year-olds in New Zealand Aotearoa on SFG.^38^ These findings weaken the argument of SFG opponents—including former UK Prime Ministers Johnson and Truss^45^—because they highlight many of those at greatest risk of using cigarettes welcome the protection from tobacco companies and their products.

A small minority, largely those with a history of tobacco use, argued against the principle of restricting choice through SFG because of its infringement on individual freedom, and to a lesser extent, the subversion of the convention of adulthood beginning at 18. This is not unexpected; similar findings were reported by some participants in qualitative studies in the United States, Singapore, Tasmania, and New Zealand Aotearoa.^10,38,46,47^ Some of these participants predicted a “backfire effect,” where young people smoke more to rebel against SFG; however, previous age-of-sale rises from 16 to 18 the United Kingdom^48^ and from 18 to 21 in the United States^6^ have reduced smoking rates. Careful communication of SFG will be required to avoid the law appearing to be a punitive measure, but instead a positive stride towards a smokefree nation. Successful tobacco control can support shifts in societal norms towards smoking law—the UK *indoor* smoking ban was relatively contentious at the time of its introduction in 2007, but now public opinion is in favor of *outdoor* smoking bans^49^—so effective implementation of the law may change negative views of SFG over time.

Many participants who were in general support of SFG still felt that, as a standalone policy, it would be insufficient to eradicate smoking in young people and strongly advocated for licensing for a smaller number of tobacco retailers. Young people’s instincts and lived experience align with research evidence; the effectiveness of age-of-sale bans for tobacco is strongly linked to the degree of enforcement and awareness of the bans^29,50^ and recent modeling for New Zealand Aotearoa projected that its repealed licensing restrictions would have resulted in an early step-change in smoking rates.^15^ Participants living in areas of higher deprivation were all very aware of shops in their area that sold underage tobacco and e-cigarette products. Governments introducing SFG policies should consider combining restrictive licensing policies, and direct resources for enforcement in areas with high youth smoking rates and lower adherence to existing age-of-sale laws to have the greatest impact on health inequalities. This is not just important for successfully lowering smoking rates, but in building trust with young people that government can deliver on its promises.

Whether e-cigarettes should be covered by SFG mandates is a topic of debate. Some of our sample supported e-cigarettes being covered by SFG and many found their omission the most illogical element of the UK government’s approach to SFG. These participant views were driven by the idea that e-cigarettes are at least as harmful as cigarettes—a view known to be growing amongst the UK public^51^—and by awareness of the far greater prevalence of e-cigarette use than tobacco use among young people in the United Kingdom. However, some current e-cigarette users were relieved to hear they would not be included, reflecting findings from a qualitative study of 15–25-year-olds with experience of dual tobacco/e-cigarette use in United States, where participants expressed disappointment that Tobacco 21 would cover e-cigarette products.^46^ The UK government had planned to ban disposable e-cigarettes and restrict marketing and availability rather than including them in the SFG, in order to retain e-cigarettes as a quit aid for adults while reducing its appeal for children.^27^ It is possible a phased approach to SFG—which first covers tobacco and subsequently e-cigarettes, when smoking prevalence drops to a pre-set level—could best achieve this balance.

### Limitations

Our study has some weaknesses. The research took place at a time when the UK policy landscape on e-cigarettes and tobacco was changing quickly. During data collection, the government announced plans to restrict e-cigarettes, including disposable e-cigarettes to be banned. Researchers asked questions in a similar manner and avoided explaining any new laws to maintain consistency across focus groups and participant perspectives; however, knowing that this law was to be introduced may have influenced some of the discussion around the inclusion or exclusion of e-cigarettes from SFG. Despite researcher efforts to promote open and frank discussion, social desirability bias and deference to perceived authority may have influenced participant answers. Our sample lacked representation from participants who identified as non-binary or as “other” genders. Seven participants from two focus groups chose to withhold their postcode; however, during focus groups, most mentioned they lived in the same areas as other participants, suggesting they lived in more deprived postcodes.

## Conclusions

These findings provide crucial evidence as to how young people, particularly those likely to be affected by the SFG policy, view its legitimacy, its likelihood of success, and its optimal implementation. Participants were largely supportive of SFG, with a minority opposed, although many felt significant efforts would be required to enforce it. Our study suggests that the support of young people can be strengthened by including them in its design and implementation, considering the context for inclusion of e-cigarettes, focusing communication on the positive SFG goal of a generation free from addiction to tobacco, and investing time and resources into enforcing retailer compliance.

## Supplementary Material

Supplementary material is available at *Nicotine and Tobacco Research* online.

ntae300_suppl_Supplementary_File_1

ntae300_suppl_Supplementary_File_2

ntae300_suppl_Supplementary_File_3

## Data Availability

The underlying thematic framework is presented in the supplementary material; raw data are restricted due to limited permissions given by participants.

## References

[1] Opazo Breton M, Gillespie D, Pryce R, et al Understanding long-term trends in smoking in England, 1972–2019: an age–period–cohort approach. Addiction. 2022;117(5):1392–1403. doi: https://doi.org/10.1111/add.15696 34590368

[2] Jackson SE, Garnett C, Shahab L, Oldham M, Brown J. Association of the COVID-19 lockdown with smoking, drinking and attempts to quit in England: an analysis of 2019–20 data. Addiction. 2021;116(5):1233–1244. doi: https://doi.org/10.1111/add.15295 33089562 PMC8436745

[3] World Health Organization. WHO Framework Convention on Tobacco Control. Geneva, 2003.

[4] 116th Congress. *Tobacco to 21 Act*. 2019. https://www.congress.gov/bill/116th-congress/house-bill/2411/text

[5] Tobacco Control Laws. Tobacco Control Laws: Sales Restrictions. https://www.tobaccocontrollaws.org/legislation/find-by-policy?step=policies. 2023. Accessed April 21, 2023. https://www.tobaccocontrollaws.org/legislation/find-by-policy?step=policies

[6] Davies N, Bogdanovica I, McGill S, Murray RL. What is the relationship between raising the minimum legal sales age of tobacco above 20 and cigarette smoking? A systematic review. Nicotine Tob Res. 2024. doi: https://doi.org/10.1093/NTR/NTAE206 PMC11847775 39234626

[7] Grube JW, Lipperman-Kreda S, García-Ramírez G, Paschall MJ, Abadi MH. California’s tobacco 21 minimum sales age law and adolescents’ tobacco and nicotine use: differential associations among racial and ethnic groups. Tob Control. 2022;31(e2):e126–e133. doi: https://doi.org/10.1136/tobaccocontrol-2020-056219 34193606 PMC8716668

[8] Macinko J, Silver D. Impact of New York City’s 2014 increased minimum legal purchase age on youth tobacco use. Am J Public Health. 2018;108(5):669–675. doi: https://doi.org/10.2105/AJPH.2018.304340 29565664 PMC5888055

[9] Silver D, Bae JY, Jimenez G, Macinko J. Compliance with minimum price and legal age for cigarette purchase laws: evidence from NYC in advance of raising purchase age to 21. Tob Control. 2016;25(3):289–294. doi: https://doi.org/10.1136/tobaccocontrol-2014-051860 25673327

[10] Lee JK, Lin L, Lim MJR, et al National tobacco control policies from the perspectives of Singapore young male adults. J Psychoactive Drugs. 2020;52(1):5–12. doi: https://doi.org/10.1080/02791072.2019.1706792 31852369

[11] Youth A, Tompkins LK, Sears CG, et al “If you are old enough to die for your country, you should be able to get a pinch of snuff”: views of tobacco 21 among appalachian youth. J Appl Res Child. 2018;8(2):2. doi: https://doi.org/10.58464/2155-5834.1334 PMC5787053 29379673

[12] Khoo D, Chiam Y, Ng P, Berrick AJ, Koong HN. Phasing-out tobacco: proposal to deny access to tobacco for those born from 2000. Tob Control. 2010;19(5):355–360. doi: https://doi.org/10.1136/tc.2009.031153 20876075 PMC2978941

[13] Berrick AJ. The tobacco-free generation proposal. Tob Control. 2013;22(suppl 1):i22–i26. doi: https://doi.org/10.1136/tobaccocontrol-2012-050865 23591500 PMC3632970

[14] van der Deen FS, Wilson N, Cleghorn CL, et al Impact of five tobacco endgame strategies on future smoking prevalence, population health and health system costs: two modelling studies to inform the tobacco endgame. Tob Control. 2018;27(3):278. doi: https://doi.org/10.1136/tobaccocontrol-2016-053585 28647728

[15] Ouakrim DA, Wilson T, Waa A, et al Tobacco endgame intervention impacts on health gains and Māori:non-Māori health inequity: a simulation study of the Aotearoa/New Zealand Tobacco Action Plan. Tob Control. 2024;33(e2):e173–e184. doi: https://doi.org/10.1136/tc-2022-057655 36627213 PMC11672025

[16] Zeng Z, Cook AR, van der Eijk Y. What measures are needed to achieve a tobacco endgame target? A Singapore-based simulation study. Tob Control. 2024;33(6):745–751. doi: https://doi.org/10.1136/tc-2022-057856 37280063

[17] Doan TTT, Tan KW, Dickens BSL, et al Evaluating smoking control policies in the e-cigarette era: a modelling study. Tob Control. 2020;29(5):522–530. doi: https://doi.org/10.1136/tobaccocontrol-2019-054951 31484800 PMC7476271

[18] Department of Health and Social Care. Modelling for the smokefree generation policy. December 1, 2023. Accessed March 8, 2024. https://www.gov.uk/government/publications/smokefree-generation-policy-modelling-report/modelling-for-the-smokefree-generation-policy

[19] Walters E, Barnsley K. Tobacco-free generation legislation. Med J Aust. 2015;202(10):509–509. doi: https://doi.org/10.5694/MJA15.00416 26021352

[20] Timberlake DS, Laitinen U, Kinnunen JM, Rimpela AH. Strategies and barriers to achieving the goal of Finland’s tobacco endgame. Tob Control. 2020;29(4):398–404. doi: https://doi.org/10.1136/TOBACCOCONTROL-2018-054779 31152117 PMC7361028

[21] Channel News Asia. Commentary: Malaysia wasted an opportunity to be bold with smoke-free law. December 2024. Accessed March 12, 2024. https://www.channelnewsasia.com/commentary/malaysia-smoking-vaping-ban-generation-legal-vape-3972256

[22] Euractiv. EU laws block Danish government’s nicotine ban. April 2022. Accessed March 12, 2024. https://www.euractiv.com/section/politics/short_news/eu-laws-block-danish-governments-nicotine-ban/

[23] McCall C. A smoke-free generation: New Zealand’s tobacco ban. Lancet. 2022;399(10339):1930–1931. doi: https://doi.org/10.1016/S0140-6736(22)00925-4 35598614

[24] Mckee M, Hopkinson NS. New Zealand reverses landmark tobacco controls. BMJ. 2023;383:e078799. doi: https://doi.org/10.1136/BMJ-2023-078799 38084499

[25] AP News. Court upholds town bylaw banning anyone born in 21st century from buying tobacco products. 2024. Accessed June 26, 2024. https://apnews.com/article/tobacco-ban-21st-century-brookline-massachusetts-e9d54d60ddfe23f9d7e06eb724f5449a

[26] Action on Smoking & Health. Renewed momentum on tobacco endgame. 2024. Accessed October 11, 2024. https://ash.org/renewed-momentum-on-tobacco-endgame/

[27] UK Parliament. Tobacco and Vapes Bill. Accessed June 26, 2024. https://bills.parliament.uk/bills/3703/stages

[28] The Labour Party (UK). Labour’s Manifesto: Build an NHS fit for the future. 2024. Accessed July 8, 2024. https://labour.org.uk/change/build-an-nhs-fit-for-the-future/#public-health

[29] Nuyts PAW, Kuijpers TG, Willemsen MC, Kunst AE. How can a ban on tobacco sales to minors be effective in changing smoking behaviour among youth?—A realist review. Prev Med. 2018;115:61–67. doi: https://doi.org/10.1016/J.YPMED.2018.08.013 30144483

[30] Aceves-Martins M, Aleman-Diaz AY, Giralt M, Solà R. Involving young people in health promotion, research and policy-making: practical recommendations. Int J Qual Health Care. 2019;31(2):147–153. doi: https://doi.org/10.1093/INTQHC/MZY113 29788085

[31] Pratt B. Engagement as co-constructing knowledge: a moral necessity in public health research. Bioethics. 2019;33(7):805–813. doi: https://doi.org/10.1111/BIOE.12591 31099899

[32] DiCicco-Bloom B, Crabtree BF. The qualitative research interview. Med Educ. 2006;40(4):314–321. doi: https://doi.org/10.1111/J.1365-2929.2006.02418.X 16573666

[33] Heary CM, Hennessy E. The use of focus group interviews in pediatric health care research. J Pediatr Psychol. 2002;27(1):47–57. doi: https://doi.org/10.1093/JPEPSY/27.1.47 11726679

[34] Guba EG, Lincoln YS. Paradigmatic controversies, contradictions, and emerging confluences. In: The Sage Handbook of Qualitative Research. 3rd ed. CA: Sage Publications Ltd; 2005:191–215.

[35] Social Research Association. Research with children and young people. 2023. Accessed October 14, 2024. https://the-sra.org.uk/SRA/Shared_Content/Events/Event_display.aspx?EventKey=RCYP301123

[36] Ahern KJ. Ten tips for reflexive bracketing. Qual Health Res. 1999;9(3):407–411. doi: https://doi.org/10.1177/104973239900900309

[37] Tong A, Sainsbury P, Craig J. Consolidated criteria for reporting qualitative research (COREQ): a 32-item checklist for interviews and focus groups. Int J Qual Health Care. 2007;19(6):349–357. 17872937 10.1093/intqhc/mzm042

[38] Hoek J, Lee E, Teddy L, et al How do New Zealand youth perceive the smoke-free generation policy? A qualitative analysis. Tob Control. 2024;33(3):346–352. doi: https://doi.org/10.1136/tc-2022-057658 36283832 PMC11474253

[39] Department of Health & Social Care. Stopping the start: our new plan to create a smokefree generation. 2023. Accessed October 13, 2023. https://www.gov.uk/government/publications/stopping-the-start-our-new-plan-to-create-a-smokefree-generation/stopping-the-start-our-new-plan-to-create-a-smokefree-generation#enforcement

[40] Michie S, van Stralen MM, West R. The behaviour change wheel: a new method for characterising and designing behaviour change interventions. Implement Sci. 2011;6(1):42. doi: https://doi.org/10.1186/1748-5908-6-42 21513547 PMC3096582

[41] Adler K, Salanterä S, Zumstein-Shaha M. Focus group interviews in child, youth, and parent research: an integrative literature review. Int J Qual Methods. 2019;18:18. doi: https://doi.org/10.1177/1609406919887274

[42] QSR International. NVivo (Version 12)[computer software]. Published online 2018.

[43] Lincoln YS, Guba EG. But is it rigorous? Trustworthiness and authenticity in naturalistic evaluation. New Dir Progr Eval. 1986;1986(30):73–84.

[44] Gale NK, Heath G, Cameron E, Rashid S, Redwood S. Using the framework method for the analysis of qualitative data in multi-disciplinary health research. BMC Med Res Methodol. 2013;13(1):1–8. 24047204 10.1186/1471-2288-13-117 PMC3848812

[45] BBC News. Rishi Sunak’s attempt to ban smoking is nuts, says Boris Johnson. 2024. Accessed October 17, 2024. https://www.bbc.co.uk/news/uk-politics-68787914

[46] Antin TMJ, Hunt G, Kaner E, Lipperman-Kreda S. Youth perspectives on concurrent smoking and vaping: Implications for tobacco control. Int J Drug Policy. 2019;66:57–63. doi: https://doi.org/10.1016/J.DRUGPO.2019.01.018 30703608

[47] Gall S, Waddingham S. *Smoking among Young People in Tasmania Report #4-Stakeholder and Youth Interviews*.; 2020. Accessed July 12, 2024. smokefreetasmania.com/wp-content/uploads/2020/09/T21-Report-4-Stakeholder-and-youth-interview-report-final-020920.pdf

[48] Beard E, Brown J, Jackson S, et al Long-term evaluation of the rise in legal age-of-sale of cigarettes from 16 to 18 in England: a trend analysis. BMC Med. 2020;18(1):85. doi: https://doi.org/10.1186/s12916-020-01541-w 32264873 PMC7140583

[49] Action on Smoking and Health. Fifteen Smokefree Years: Public support in England for measures to reduce the harm of smoking. 2022. 2022. Accessed October 16, 2024. https://ash.org.uk/resources/view/fifteen-smokefree-years-public-support-in-england-for-measures-to-reduce-the-harm-of-smoking

[50] DiFranza JR. Which interventions against the sale of tobacco to minors can be expected to reduce smoking? Tob Control. 2012;21(4):436–442. doi: https://doi.org/10.1136/TOBACCOCONTROL-2011-050145 21994275

[51] Jackson SE, Tattan-Birch H, East K, et al Trends in harm perceptions of E-cigarettes vs cigarettes among adults who smoke in England, 2014-2023. JAMA Netw Open. 2024;7(2):e240582–e240582. doi: https://doi.org/10.1001/JAMANETWORKOPEN.2024.0582 38416490 PMC10902732

