# Supplementary material for: Perceptions of Children and Young People in England on the Smokefree Generation Policy: A Focus Group Study
Source: Nicotine Tob Res. 2024 Dec 17;27(6):1066–72. doi: 10.1093/ntr/ntae300 (PMC12095808; doi:10.1093/ntr/ntae300)
Supplement: ntae300_suppl_Supplementary_File_1 [file ntae300_suppl_supplementary_file_1.pdf]

Focus group schedule for children and young people on raising the age of sale of tobacco in the UK

The questions are mapped across to the COM-B framework: C – capability, O – opportunity, M – motivation.

Before interview

Explain purpose of study. Go through the participant information sheet with participants. Reiterate points about consent, confidentiality, safeguarding, recording, treatment of data and withdrawal. Remind them that everything said is confidential and should not be repeated outside of the room.

Interviewer: Do you have any questions about any part of the study?

Personal experiences of smoking/vaping

1. Does anybody know someone who smokes or vapes? **Probe: who, do they know how they started?**

2. Has anybody tried smoking or vaping themselves? **Probe: how did you start?**

3. What do you think are the main reasons that young people start smoking? **Probe: peer pressure, (stress/boredom), control/rebellion?**

4. How do you or people you currently know get hold of cigarettes or vapes? **(O, C)**

Concept of smokefree generation law (SFG)

5. Do you know what the current age of sale of cigarettes is in the UK? **(C)**

6. Do you know what the current age of sale for vapes/e-cigarettes is in the UK? **(C)**

Explain that it is currently 18. Explain that the Government has a plan to change this in England. Shops and other sellers could be banned from ever selling to anyone born after a certain year e.g. to children who are currently 14 (those born in 2009 or later). So, even when 14-year-olds reach 18, 19, 20, they wouldn't be able to buy cigarettes. Individuals could not be criminalised or punished for buying cigarettes – only shops could be punished for selling them. This would raise the age of sale one year every year. The age of sale for vapes would remain at 18.

7. Do you understand the law? **(C)**

8. What are your thoughts on this idea if it were introduced?

9. Think about people who currently smoke but want to quit. What would this measure have meant for them? (**O, C, M**)

10. What are your thoughts on whether this could stop young people from trying a cigarette in the first place? (**O, C) Probe: How might they get hold of a cigarette?**

11. What are your thoughts on whether this could stop young people from trying an e-cigarette in the first place? (**O, C, M)**

<u>Implementation of SFG</u>

12. Do you have any other thoughts around how age of sale laws might work?

13. How might these kinds of new laws affect how young people think about cigarettes? **(M)**

14. How might these kinds of new laws affect how young people think about vapes? **(M)**

Probe: Do you think it would change how young people talk about cigarettes/vapes with their friends? (M)

15. How might this law change how cigarettes/vapes are portrayed in the media or social media you use? **(M)**

Probe: Are there any other negatives or benefits to the law?

16. What would it be like to be part of a generation where nearly no-one smokes? **(M)**

What do you think of the following ideas?

17. The Government sets up a group of young people to advise them on how to implement the Smokefree Generation idea. **(O, C, M)**

    PROBE: What sort of young people should be on the group? How much influence should they have?

18. The Government introduces licenses for retailers who still sell tobacco to young people. If they are caught selling tobacco to those underage, they lose their license. **(O, C)**

    PROBE: How effective would that be?

19. The government runs a marketing campaign to inform people about the age of sale law. **(C, M)**

PROBE: What would be the most important messages? Who should communicate these? Should there be different messages for different groups?

20. Are there other rules, laws or support, if any, do you think should be put in place at the same time as new laws on age of sale? **(O, C, M)**

21. Do you have any other thoughts you'd like to share?

Thank participants for their participation and explain the next steps for the research.
