# Supplementary material for: Perceptions of Children and Young People in England on the Smokefree Generation Policy: A Focus Group Study
Source: Nicotine Tob Res. 2024 Dec 17;27(6):1066–72. doi: 10.1093/ntr/ntae300 (PMC12095808; doi:10.1093/ntr/ntae300)
Supplement: ntae300_suppl_Supplementary_File_2 [file ntae300_suppl_supplementary_file_2.docx]

**Overview of public involvement in project**

Three separate groups of public advisors aged 12-21 (n = 20) provided advice on inclusion criteria, recruitment methods and the topic guide. These groups were drawn from two pre-existing youth forums of secondary school-aged children in England, one educational project designed to give young people for whom mainstream education was not fulfilling their needs. Finally, two young adults aged between 18 – 21 with experience of tobacco use provide public contributions to the overarching RAISE research project that this study forms part of. The pre-existing youth forums receive existing forms of compensation from their parent organisations, but 10 GBP shopping vouchers were provided to participants from the educational project and the two young public contributors were paid 25 GBP per hour for their contributions.

The pre-existing youth forums (n = 5 and n = 6) were first consulted on during one of their pre-scheduled meetings on the scope of the research, with age-of-sale deemed to be an important topic to study and researching the perspectives of young people deemed an important priority within this, second only to establishing links between age-of-sale policies and overall health outcomes. They suggested routes into recruiting participants, including the importance of fitting it into young people’s already existing activities rather than holding it as a separate activity, and holding focus groups rather than individual interviews, advice we followed.

Next, young people from the educational project (n = 7) and the two public contributors were consulted separately before the study. They provided feedback on participant information sheets and topic guides, helping simplify language and make questions more explicit. Young people from the educational project also suggested recruiting those aged 11+, not just those aged 13+ as was originally planned. We acted on this feedback to widen the inclusion criteria.

They also provided advice helping participants feel at ease, and ways to encourage participants to be honest, with particular focus on ensuring young people knew no repercussions could come from school or home if they divulged use of cigarettes and/e-cigarettes. We built this into the focus group data collection process by ensuring there was plenty of time pre-focus group to discuss the process and the safeguards put in place around confidentiality.

We express our sincere thanks to all public participants for their invaluable help.
