# Supplementary material for: Perceptions of Children and Young People in England on the Smokefree Generation Policy: A Focus Group Study
Source: Nicotine Tob Res. 2024 Dec 17;27(6):1066–72. doi: 10.1093/ntr/ntae300 (PMC12095808; doi:10.1093/ntr/ntae300)
Supplement: ntae300_suppl_Supplementary_File_3 [file ntae300_suppl_supplementary_file_3.pdf]

	Escape from harmful addiction	Impact of SFG is not guaranteed	Offer protection and a voice
**1 : Focus group #1 (12 - 14)**	Acutely aware of risks of smoking, addictiveness of smoking, and how this has affected their parents.<br><br>P1.4 (F13, NS/NV) "My parents are trying so hard to get out of smoking, but I think since they've done it from such a young age, they can't really get off it and it's affecting them.... because (they) wanna grow older and see us grow up and see us have loads of birthdays and see us be able to become successful".<br><br>P1.6 (F14,TS/RV) "It's a really addictive chemical nicotine. It's like it's not something you can just like, stop. Like they really hard to like hide all the cigarettes and stuff from us like. Like, you know, they just don't really want us to see them doing it to them because they obviously don't want us."<br><br>P1.2 (F14, NS/NV) "We, actually, me and my brother, we had a conversation with my mum ages ago, because we were like really worried about it. And she said that she was gonna try and stop, but she really hasn't. And it must be difficult because it's not something that she really talks to us much about."<br><br>Two disagreed with SFG on point of individual rights, others strongly in favour of the smokefree vision.<br><br>P1.4 (F13, NS/NV) I think it's a nice thing to think about when people look back into the past they'll think, wow, these people stopped smoking.<br><br>P1.3 (M13, NS/NV) Plus, it's their body, kind of. It's not really affecting anyone else. It's just your choice of smoking, really.<br><br>One participant believed that vapes should be banned too. Another agreed with the government approach of placing other restrictions on vapes.	This group held mixed views. Some very positive views on how SFG could prevent young people from starting smoking at their most vulnerable to addiction. Others were worried about the possibility of it boosting illegal tobacco. Some argument that most young people were interested in vapes, not tobacco, which made the law less relevant.<br><br>P1.4 (F13, NS/NV) "It stops them at a young age when they're trying new things and then. If they do it any later, they've already decided to try it and it's too late to stop them.<br><br>P1.6 (F14,TS/RV)) "It's just gonna cause more illegal activity because there's more people giving them illegally. So there's no point. I don't think there's any point of doing anything with the cigarettes or the vapes because it's just going to cause more havoc for the government."	Two participants were very supportive of introducing licenses and reducing the number of shops that can sell vapes and tobacco. One did not think it would prevent shops from acting<br><br>P1.6 (F14,TS/RV). "Yeah, because the one is ridiculously close and it's not only next to our high school, but it's next to a primary school. Two primary schools really. There was generally strong support for bringing younger people in to advise."<br><br>There was some disagreement about how to get message across - some believing health impact should be front and centre, others that the smokefree vision should be centred.<br><br>P1.6 (F14,NS/TR) "Actually I the actual message is to get across that that mortality isn't the most important thing this is gonna affect you for the rest of your life. So cancer now, next year and then it'll get worse, you won't die in your sleep."<br><br>Participants raised the idea of considering people buying vapes/cigarettes through social media and fake ID.
**2 : Focus group #2 (15 - 18)**	General support for the idea philosophically across the group on the basis of stopping ill health. One strong moral objector on the basis of it restricting freedom.<br><br>P 2.7 (M18, NS/TV). "It's a good idea. Because young people can get addicted easily. At the age of 18, it's too hard for them to make a decision. So I think it's good for them not to smoke at all.<br><br>Didn't believe vapes should be included too. Some varying views on how vapes should be regulated, from restricting shops that can sell them to taxes to banning foreign imports.<br><br>P2.6. (F15, NS/RV) "The kids are like, oh it's cotton candy - that should be banned. And then these *points to refillable vape* should be in chemists and proper actual stores."	Described as a smart idea by many in the group. One said they would support if the idea has evidence based backing. One participant felt the idea was counterproductive and that people will still get hold of them despite the new law, drawing on experiences of parents and older friends buy vapes for some participants. Others buy directly from local shops.<br><br>P2.1. (F17, TS/RV) "You're gonna have all these people trying their hardest to get them, get a bunch of crates in so they can also sell them separately...the hype, it's actually going to become more".	Some varying views on how vapes should be regulated, from restricting shops that can sell them to taxes to banning foreign imports.<br><br>P2.6. (F15, NS/RV) "The kids are like, oh it's cotton candy - that should be banned. And then these *points to refillable vape* should be in chemists and proper actual stores."

**3 : Focus group #3 (13 - 16)**	Uncertainty on current age of sale of tobacco and e-cigarettes - guesses for 16, 17 and 18.<br><br>Several reports of older peers pressing them to try either cigarettes or vapes. Many tried cigarettes before vapes.<br><br>P3.4 (M14, TS/RV) "When I was ten, my sisters ex boyfriend. Yeah, he got me, he approached me to smoke cigarettes and start vaping and then I got addicted from there."<br><br>One participant articulated that it was very hard to stop smoking and vaping. Being surrounded by others who smoke or vape increases the difficulty.<br><br>P 3.6 (M14, NS/RV) But then when you think about it, it's like don't bother with it because then it's gonna hurt me even more, don't die from it. Like my grandmother died from cancer about 5 months ago, I keep on worrying about that, but then like I keep on forgetting about it. But like I go on and off, but then it hurts, to think.<br>Some participants supportive of SFG and the way it attempts to reduce harm.<br><br>A couple of the group described "not caring" about the law because they do not desire to buy cigarettes. One participant was not supportive of TFG in principle, mainly due to views on it restricting choice.<br><br>P3.4 (M14, TS/RV) "18 is like the legal age that you're an adult."<br><br>P3.3 (14F, NS/RV) "I'm not bothered because I'm not trying to get a pack of cigarettes."	Some participants thought that SFG would lead to greater illicit activity and purchasing. Others thought it could be part of a denormalisation process.<br>Perception that both vapes and cigarettes are easy to access in their local town, both through proxy purchasing and direct retail. Unsure that SFG will make it harder to get hold of cigarettes<br><br>P3.4 (M14, TS/RV) "I think it would be pretty easy. [Town] is just [town]! I'd probably just go to bossman's shop."<br><br>P 3.3 (M14, NS/RV) "It's like if I were one of the only ones to do it, and then people probably were looking at me. I would probably think, "What am I doing to myself and like is this good for me?"	Did not want communication to be "finger wagging". Did not express views on other policies that could be introduced when asked.
**4 : Focus group #4 (14 - 16)**	All started vaping after hanging out with other people, often those who were older.<br><br>Agreement that cigarettes are not appealing to themselves personally or the wider population of young people aged 14-16.<br><br>P4.1. (F14, NS/RV) "Because (smoking cigarettes) is disgusting."<br><br>P 4.3 (M14, NS/RV) "The way I see it is, more people of my generation have already stopped smoking cigarettes, yeah? Here in the UK, most kids don't smoke in there."<br><br>Uncertainty on the harms caused by e-cigarettes.<br><br>P.4.3 (M14, NS/RV) "It depends a lot on research they're looking to. Some sites could say that it's worse. Sometimes it says the other is worse It would depend on the view that you actually look into it, because different people have different opinions. Teacher could say, you know, smoking is worse. So it's just opinions."<br><br>Know that age of-sale is 18 for vapes but some participants guessed that the age of sale is set to 21 for cigarettes.<br><br>Didn't have particularly strong views on SFG. Some support that the same broader restrictions that apply to cigarettes should apply to vapes - packaging, placement, pictures, but not that it should necessarily be part of SFG.<br><br>P 4.2 (F16, NS/RV) I think corner shops shouldn't be able to sell vapes, because you have more chance to get served. Whereas normal vape shops, or Asda, you get IDed.	Afte some initial worries about illegal tobacco, the group came to the conclusion that those who have already started trying cigarettes will keep getting hold of them through older friends and family, but it would work as prevention for those who haven't tried cigarettes yet.<br>Agree that vapes are bigger issue. Older people buy vapes for this group, but they also access from local shops, estimating that 1 in 3 shops would sell to them.<br><br>P 4.3 (M14, NS/RV)"People our age, they probably still want them because if you're… depends how addicted you are to it. If you're really addicted, you'll try and get hold of them. If not, you won't".<br><br>More than one participant states that vapes and cigarettes are substitution products.<br><br>P 4.2 (F16, NS/RV) "Probably when vapes get banned, probably will try a cigarette."<br><br>P. 4.1(F14, NS/RV) It'll push more people towards vapes instead of cigarettes because they don't have the other option.	Group makes case for more funding for enforcing shops selling the law, because they aware the proportion who avoid.<br><br>Believe that a license should be introduced for selling nicotine products.<br><br>P 4.2 (F16, NS/RV) "I'd probably increase fines on the shops, we can take down the whole thing. Yeah. The licence would be good, because little kids can get served and then it's encouraging them to vape."<br><br>One participant mentioned that health problems should be front and centre of communications.

**5 : Focus group #5 (18 - 20)**	Tobacco initiation after spending time out with a group of kids who had started smoking cigarettes (vapes were rare amongst their demographic at school).<br><br>Participants discussed whether younger are really addicted to vapes, because they do it infrequently. One participant believes cigarettes are not good, despite smoking them occasionally - they are just an option when one is drunk or needs a nicotine hit.<br><br>P 5.3: (F18, TS/RV) I don't think they're addictive because they don't do it at home, except here they're doing this.<br>P 5.2: (F20, TS/RV) They're doing it to look cool.<br><br>Understand the health harms of smoking tobacco, but sceptical of the idea that vaping is a lot safer than cigarettes.<br><br>P 5.2 (F20, TS/RV) "Thing is with vaping, it's still quite new. So you don't know the long-term effects of it?"<br><br>Two participants supported the idea, on the basis that cigarettes cause extreme damage and addiction are not "needed" to enjoy life.<br><br>One participant was initially against SFG on the basis it denies pleasure, but later appeared to be softening her opposition.<br><br>P5.3 (F18, RS/RV) "But still, on a night out. They're never gonna experience that, bless their little cotton socks. Like you only live once, you might as well experience everything.2<br><br>Participants expressed that vaping helps with anxiety to some extent, tastes nice. One participant said she would "die" without vapes.<br><br>However, there was strong agreement that it is logical to ban vapes as well as cigarettes.<br><br>P 5.2 (F20, TS/RV). "I don't know. I feel like they're both as bad as each other, so if they're going to do one, they may as well do the other. So it's like you're saying, that's bad for you, but it's fine you can do this still. It doesn't make sense."	Agreed it would have been harder to get hold of cigarettes when young as could no longer rely on those slightly older to buy. Believe it is likely to work. Older friends were the ones who used to buy cigarettes and vapes for this group before they could be legally sold them.<br><br>P. 5.1. (F20, RV/RS) "If (cigarettes) are not as easy to buy, no-one's going to do it, too much hassle."	One participant was relatively sceptical of the idea of a license, articulating that the alcohol license does not appear to be successful in preventing underage sales of alcohol. Others were more supportive of idea.<br><br>Believe shops will be canny about getting round the rules - will advertise through word-of-mouth and be at pains to conceal sales.<br><br>P 5.2 (F20, RV/RS) P. 5.1 : "You get fined or closed down. Simple."
**6 : Focus group #6 (12 - 15)**	Influenced by family members who already smoked or vaped, and was given or stole cigarettes. Significant interest was displayed in other forms of nicotine, like nicotine pouches. Some positive views of vapes put forth as a stress reliever.<br><br>P 6.3 (F14, TS/RV) "I was stressed. So one of my older like cousins in year 11, Yeah, they just told me to like take a puff. So I did. And I did feel better. So I just started vaping."<br><br>Three participants reported negative feelings towards the anti-smoking posters in school, because they feel like the messages are coming from the teachers, who they do not respect.<br><br>Divergent estimates that the age of sale was 16 and 18 for both vapes and cigarettes<br><br>The group was divided on principle of SFG. Many didn't mind about the law as they are not interested in cigarettes. Some supported on the idea it restricts the damage caused by a dangerous product, others wanted to be able to keep vaping and smoking and are against it.<br><br>P 6.5. (F14, RS/RV) "I'm not gonna lie to you when I see like, people, not even 11, seven-year old, 7 year olds… It's sad, so sad."	Some participants believed it would work due to fear.<br><br>P 6.2 (M15, FS/RV): "Yeah, it's gonna be harder because they're gonna be more scared about that. It's scary when you go to buy a vape that's illegal."<br><br>Others believed that shopkeepers would continue selling and it would have little impact. Also raised impact on mental health for those having to forgo nicotine.<br><br>This group find it fairly easy to get hold of cigarettes and vapes, and get it from local shops. One participant pays extra to ensure that the shopkeeper will sell to them.<br><br>P6.4 (F13, TS/RV). "Actually the first time I tried it, it was a fag because my mum, my mum was like she told me to go get her fags and then I was like OK then."<br><br>Determination amongst much of the group to get round a	Group one of the least likely to believe the law will change anything in the way local shops operate, who they believe will do anything for money. Some participants feel an affinity with the shopkeepers who sell to them. Two participants believe very close enforcement or restricting sales to certain shops will help.<br><br>P 6.2:(M15, FS/RV) In [area] you can buy anything. Yeah, you can even buy alcohol. Just go to shop, say I want that, they give it to you. They don't ask for nothing. I recommend you just shut down every shop in [area].<br><br>In terms of communication, there is strong agreement they don't want to feel lectured to, Don't identify with teachers who run assemblies on the topic who have had different lives to them.

	P 6.4. (F13, TS/RV). Yeah. I'm not really into fags. It wouldn't really bother me.<br><br>Divided opinions on the relationship with SFG and e-cigarettes. Some find it relieves stress and do not want to quit. Some want to stop and hate the feeling of addiction. One participant made the case for banning foreign vapes.<br><br>P 6.3: (F14, TS/RV).: Like if I really wanted to, I could stop. I did ask could. I just don't want, can't be arsed.<br><br>P 6.2 (M15, FS/RV) I would recommend don't start. I would recommend don't start. I tried quitting like 30 times. Couldn't.... They ruined my life. I've had so many problems since I started smoking.	disposable vapes ban, either moving to rechargeable vapes or even supplying vapes to those who are younger than them, but not the same determination to avoid SFG.<br><br>P 6.3: (F14, TS/RV). No I don't want to stop (vaping).	P 6.3: (F14, TS/RV). "Like they haven't gone through what we have. Yeah. You know, like their school was different was they don't get the stress and stuff. So most of the reasons people smoke anyways because of stress."
**7 : Focus group #7 (17 - 21)**	Relate portrayal of cigarettes and vapes as being from further back in the past. They see little imagery in today's traditional or even in today's social media.<br><br>Agree that pressure from older family/friends key driver. For older participant, cigarettes was first experience with nicotine product.<br><br>Agreement amongst participants that cigarettes no longer hold appeal amongst teenagers compared to vapes.<br><br>P7.1 (M17, NS/NV) "There's kind of more of a culture of younger people saying it's not cool. It's gross."<br><br>P 7.2 (F21, RS/RV) "A lot of the vape generation… I don't think they'll ever go to cigarettes."<br><br>Agreement that vaping is riskier than many acknowledge and the long-term effects remain unknown.<br><br>P 7.2 (F21, RS/RV) "And then you have vaping like it's not been around for that long. Yeah, you don't know what situation they're gonna be in when they reach that age of like 70? Because it's just - we haven't got there yet."<br><br>Correct knowledge that 18 is age of sale for all products.<br><br>Provided some views for and against it on ethical principles. Both participants are for the principle of using it to restrict smoking but want to make sure the younger generation don't see it as unfair. However, they had strong views that vapes should be included as well as cigarettes if the policy goes ahead.<br><br>Did not describe in detail personal experience in how smoking and vaping have shaped lives. One participant is a social smoker and vaper.	Agree that SFG will help prevent many people from trying their first few cigarettes but is unlikely to stop those who might buy e.g. cannabis or have already developed a nicotine addiction. Underage access for this group was either through stealing cigarettes or through older siblings/friends.<br><br>P 7.2 (F21, RS/RV) "I think that while for some people who are actually a first buyer, they'll look if they can get cigarettes, they see they can't and they'll just give up on the whole idea."	Wary that there are many ways around the law for local shops and wary that companies might adapt to new laws to entice new smokers. Both participants advocated for education, limits on number of shops that can sell, and licenses.<br><br>Agree that vivid imagery of health impacts of smoking required. Some disagreement over whether the costs to the NHS should be included in public communication.<br><br>P7.1 (M17, NS/NV) "I think that (youth involvement) is a really good and important idea because I think it's important that young people can sort of feel they are the smoke free generation. "
